# Incidental [^68^Ga]Ga-DOTATATE uptake in bone metastases of breast carcinoma in a patient with a secondary carcinoid tumor

**DOI:** 10.1186/s13550-026-01462-4

**Published:** 2026-06-11

**Authors:** Jan Kemle, Sabin G. Pop, Alexander Sauter, Rafael Pereira, Tilly Nothhelfer, Gad Singer, Cornelia Leo, Irene A. Burger

**Affiliations:** 1 Department of Nuclear Medicine, Kantonsspital Baden, Partner hospital for research and teaching of the Medical Faculty of the University of Zurich, Baden, Switzerland; 2 Department of Radiology, University Hospital Tuebingen, Tuebingen, Germany; 3 Department of Nuclear Medicine, University Hospital Tuebingen, Tuebingen, Germany; 4 Department of Oncology, Kantonsspital Baden, Baden, Switzerland; 5 Department of Pathology, Kantonsspital Baden, Baden, Switzerland; 6 Department of Gynecology, Kantonsspital Baden, Partner hospital for research and teaching of the Medical Faculty of the University of Zurich, Baden, Switzerland; 7 Department of Nuclear Medicine, University Hospital Zurich, University of Zurich, Zurich, Switzerland; 8 Department of Health Sciences and Technology, ETH Zurich, Zurich, Switzerland

**Keywords:** Invasive lobular breast cancer, Somatostatin receptor 2, Bone metastases, Pulmonary carcinoid, [^68^Ga]Ga-DOTATATE PET/CT

## Abstract

**Background:**

Lobular breast cancer often shows only low [^18^F]FDG uptake, limiting the sensitivity to detect metastases. Alternative tracers, targeting tumor activated fibroblasts or estrogen receptors, can offer higher accuracy. Also, Somatostatin Receptor 2 (SSTR2) is significantly expressed in some breast cancers, particularly in estrogen receptor rich tumors. Typical pulmonary carcinoids also have low [^18^F]FDG accumulation, but approximately 63% of well-differentiated lesions express SSTR2.

**Case presentation:**

We report the case of a 66-year-old female patient with lobular breast cancer and a coincidentally discovered typical carcinoid of the lung. On subsequent [^68^Ga]Ga-DOTATATE PET/CT to stage the carcinoid tumor, two SSTR2 positive focal lesions in the bones were present, without [^18^F]FDG uptake but with mild sclerosis, atypical for metastasis of carcinoids. Biopsy confirmed osseous breast cancer metastases.

**Conclusion:**

SSTR2 expression in normal breast tissue and breast carcinoma has been described previously. However, the case underscores the importance of awareness of atypical presentations of cancer within a multi-tracer approach, whereby receptor profile and morphology must be carefully integrated.

**Supplementary Information:**

The online version contains supplementary material available at 10.1186/s13550-026-01462-4.

## Background

Lobular breast cancer is known to demonstrate low [^18^F]-FDG uptake in some cases, limiting the sensitivity for PET/CT in the detection of metastases [1]. Therefore, occult lesions on [^18^F]-FDG PET/CT have to be searched with contrast enhanced CT or MRI [2, 3]. Alternative PET tracers, including the fibroblast-activation protein inhibitors (FAPI) or targeting the estrogen receptors (ER) are under investigation to improve accuracy in staging lobular breast cancer [4, 5]. However, widespread availability is still limited for both alternatives. First preclinical tests suggested that agonistic somatostatin receptor 2 (SSTR2) ligands (such as [^68^Ga]Ga-DOTATATE, [^68^Ga]Ga-DOTATOC or [^111^In]In-DOTA-JR11), might provide higher sensitivity and specificity, in particular, in ER-rich tumors [6, 7]. Early clinical investigations suggested a potential role of [^99m^Tc]Tc-Octreotide imaging [8] and subsequent clinical investigations confirmed the potential use of [^68^Ga]Ga-DOTATATE to image ER-rich breast cancer [9, 10]. One study even suggested that [^225^Ac]Ac-DOTATATE might be a promising therapeutic option for some patients with SSTR2 positive breast cancer [10].

[^68^Ga]Ga-DOTATATE is typically used in imaging neuroendocrine tumors (NET), arising for the gastrointestinal tract or the lungs since these lesions have low [^18^F]-FDG-accumulation [11, 12]. Well-differentiated pulmonary carcinoids have up to 63% high SSTR2 expression on [^68^Ga]Ga-DOTATATE PET/CT^11^.Therefore, the low [^18^F]-FDG-uptake in a pulmonary lesion could be compatible with a carcinoid as well as metastasis from a lobular breast cancer and biopsy is needed. If then [^68^Ga]Ga-DOTATATE PET/CT is done to stage the pulmonary carcinoid, a positive lesion could still represent a metastasis from lobular breast cancer. This is important to know, since pulmonary carcinoids are often incidental findings in patients undergoing chest CT, with over 40% of the lesions incidentally discovered [13] and around 43% then staged with SSTR2 targeting imaging [14].

## Case presentation

**Fig. 1:**
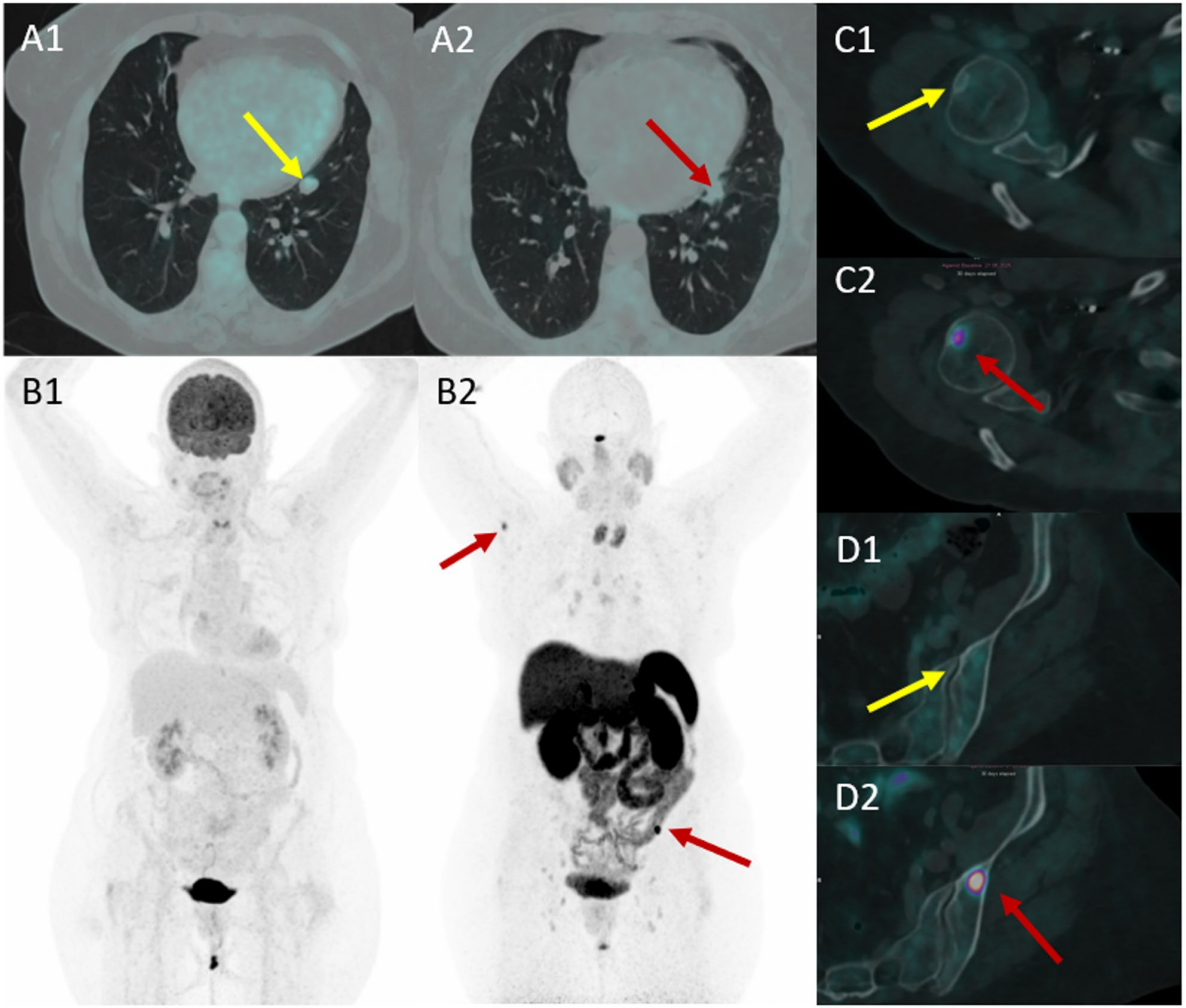
66-year-old woman after resection of a lobular carcinoma of the right breast. [¹⁸F]FDG PET/CT **(A1)** - axial fused image, the paracardial lesion (yellow arrow) has low metabolic activity. Biopsy showed a typical carcinoid (NET G1, Ki-67 5%, Estrogen receptor-negative). For staging of the pulmonary carcinoid, a [⁶⁸Ga]Ga-DOTATATE PET/CT was performed and the axial fused image **(A2)** showed only faint somatostatin receptor 2 (SSTR2) expression (red arrow). Whole-body maximum intensity projection of [¹⁸F]FDG PET/CT **(B1)** shows no increased uptake in the bones. However, on [⁶⁸Ga]Ga-DOTATATE PET/CT **(B2)** two bone lesions with intense [⁶⁸Ga]Ga-DOTATATE uptake were detected (red arrows). On axial fused [¹⁸F]FDG PET/CT (**C1/D1**) sclerotic changes, without [¹⁸F]FDG uptake were seen (yellow arrow), where fused [⁶⁸Ga]Ga-DOTATATE PET/CT showed strong SSTR2 expression (SUVmax 10.2) (red arrow) in the right humeral head lesion (**C2**) as well as in the left iliac lesion (**D2)**. The iliac bone lesion was biopsied and a SSTR2 positive bone metastases of the lobular breast cancer confirmed

We present the case of a 66-year-old woman with a history of lobular carcinoma of the right breast (ER 100%, HER2-negative, Ki-67 20%) who underwent breast-conserving surgery in March 2025. CT imaging performed for radiotherapy planning revealed a pulmonary nodule with 17 mm in the left lower lobe. To rule out pulmonary metastasis a [^18^F]-FDG PET/CT was performed and showed only minimal metabolic activity in this lesion (Fig. 1). Based on the low [^18^F]FDG accumulation in a pulmonary nodule, a metastasis from a lobular breast cancer could not be ruled out, however primary pulmonary tumor was possible as well. Therefore, a bronchoscopy with biopsy was recommended and showed pulmonary carcinoid, without evidence for breast cancer metastasis (NET, G1, Ki-67 5%, synaptophysin and chromogranin positive, ER-negative), consistent with the known low [^18^F]-FDG avidity of typical carcinoids. Subsequent [^68^Ga]Ga-DOTATATE PET/CT for staging of the carcinoid lesion demonstrated intense SSTR2 uptake in two bone lesions in the left iliac bone and right humeral head, whereas the pulmonary nodule showed only faint uptake. The bone lesions showed mild sclerosis on CT. Given that strong SSTR2 positive bone metastasis arising from a SSTR2 negative primary tumor would be unusual, as well as primary sclerotic bone lesions are not typical for a pulmonary carcinoid, a biopsy of the iliac lesion was recommended. The iliac lesion was confirmed to be metastatic lobular breast cancer.

Based on these findings, the patient started on endocrine therapy with letrozole and the CDK4/6 inhibitor abemaciclib, with partial response on subsequent [^68^Ga]Ga-DOTATATE of both bone lesions. The pulmonary carcinoid remains under surveillance.

## Conclusion

This case illustrates the crucial role of a molecular multi-tracer imaging approach in complex oncological scenarios. SSTR expression in normal breast tissue and breast carcinoma has been described in the literature. Although SSTR2 expression is often interpreted as indicative of neuroendocrine tumors, recognizing that SSTR2 expression in bone metastases from breast cancer can be an incidental yet highly useful finding helps guide patient management. Ultimately, knowledge about morphological patterns and histopathological workup in unclear cases remains essential to guide therapy and avoid misclassification of lesions.

## Supplementary Information

Below is the link to the electronic supplementary material.

Supplementary Material 1.

## Data Availability

Data are available upon reasonable request to the corresponding author.

## Abbreviations

ER: Estrogen receptor
FAPI: Fibroblast-activation protein inhibitors
SSTR2: Somatostatin receptor 2
NET: Neuroendocrine tumor
