# Supplementary material for: Incidental [68Ga]Ga-DOTATATE uptake in bone metastases of breast carcinoma in a patient with a secondary carcinoid tumor
Source: EJNMMI Res. 2026 Jun 11;16:129. doi: 10.1186/s13550-026-01462-4 (PMC13481949; doi:10.1186/s13550-026-01462-4)
Supplement: Supplementary file 1 [file 13550_2026_1462_MOESM1_ESM.docx]

Reviewer #1: EJRE-D-26-00140

SSTR2 positive bone lesions: not pulmonary carcinoid but breast cancer metastasis. Understanding complex disease with a multi-tracer approach.

Comments

This is a case report aiming to emphasize the complex nature of breast carcinoma and SSTR2 expression in various neoplastic tissues. There are many research papers available in the literature since early 1990's showing the availability of SSTR receptors in breast tissue and breast carcinomas. The findings are not new in this respect.

It is however a teaching case for the novice reader to emphasize the overlapping benefits of integrated imaging through the incidental uptake of DOATATE in bone metastases which possibly changed the management of this patient. The authors should revise their text accordingly emphasizing that SSTR availability in normal breast tissue and breast carcinoma is well known and the uptake in bone mets are incidental yet useful in the patient management.

A1: We agree and added that SSTR2 expression in breast cancer has been documented in the past and tried to express this in the first paragraph of the Background: First preclinical tests suggested that agonistic somatostatin receptor 2 (SSTR2) ligands (such as [^68^Ga]Ga-DOTATATE, [^68^Ga]Ga-DOTATOC), might provide higher sensitivity and specificity, in particular, in ER-rich tumors^6^. Subsequent clinical investigations confirmed the potential use of [^68^Ga]Ga-DOTATATE to image ER-rich breast cancer^7,8^

We found 2 more original papers looking into SSTR2 and breast using 111In and 99mTc based imaging and added them to the background. And added a sentence to the conclusion accordingly. “SSTR2 availability in normal breast tissue and breast carcinoma is known.”

Also, the paper has some typos which need to be corrected before acceptance for publication:

Page 2, line 12: " ... estrogen receptors rich...." Please correct to ".....estrogen receptor rich....".

A2: Thank you for spotting this typo. The text was corrected.

Page 3, line 28: "....have in up to 63% high SSTR2 expression...." Please correct to "..... have up to 63% high SSTR2 expression...."

A3: We have corrected the phrasing to "have up to 63% high SSTR2 expression" in the revised manuscript.

Page 4, line 1: "...negative). Consistent with the known low [18F]-FDG avidity of typical carcinoids...." Please correct to "....negative), consistent with the known low [18F]-FDG avidity of typical carcinoids...."

A4: The text has been corrected

Page 4, line 17: ".....abemaciclib, with good partial response on subsequent...." Please correct to "....abemaciclib, with partial response on subsequent.....".

A5: The text was corrected.

In addition, please choose a "hot iron" color on fusion images to better demonstrate the focal uptake of DOTATATE fused on BW CT images.

A6: As I appreciate, that color selection according to the personal preference is desired, to our knowledge the EANM guidelines are not recommending anything specific. The statement is: ***“Color Scale Selection:*** *While "PET-rainbow" (a nonlinear scale) is commonly used, and sometimes associated with increased lesion detection in specific studies, the selection of color scale is often left to the reader's discretion, as scientific evidence comparing them is limited. However, the key requirement is consistency between initial and follow-up scans.”*

Therefore, I’d like to stay with the selected warm metal scale, that has the advantage of still visible CT in the back and good lesion discrimination without a rim (as is the case for the perfusion scale).

Finally, please revise the title for a smoother read: "Incidental Ga-68 DOTATATE uptake in bone metastases of breast carcinoma in a patient with carcinoid tumor"

A7: We agree that the proposed title reads much smoother and reflects the message of this teaching case. We have changed the title.
